# A Structural Perspective on the Regulation of Human Single-Stranded DNA Binding Protein 1 (hSSB1, OBFC2B) Function in DNA Repair

**DOI:** 10.1016/j.csbj.2019.03.014

**Published:** 2019-03-28

**Authors:** Teegan Lawson, Serene El-Kamand, Ruvini Kariawasam, Derek J. Richard, Liza Cubeddu, Roland Gamsjaeger

**Affiliations:** a School of Science and Health, Western Sydney University, Sydney, Locked Bag 1797, Penrith, NSW 2751, Australia; b School of Life and Environmental Sciences, University of Sydney, NSW 2006, Australia; c Genome Stability Laboratory, Cancer and Ageing Research Program, Institute of Health and Biomedical Innovation, Translational Research Institute, Queensland University of Technology, Woolloongabba, Queensland 4102, Australia

**Keywords:** hSSB1, OBFC2B, SSBs, DNA repair, NMR, OB domains

## Abstract

Single-stranded DNA binding (SSB) proteins are essential to protect singe-stranded DNA (ssDNA) that exists as a result of several important DNA repair pathways in living cells. In humans, besides the well-characterised Replication Protein A (RPA) we have described another SSB termed human SSB1 (hSSB1, OBFC2B) and have shown that this protein is an important player in the maintenance of the genome. In this review we define the structural and biophysical details of how hSSB1 interacts with both DNA and other essential proteins. While the presence of the oligonucleotide/oligosaccharide (OB) domain ensures ssDNA binding by hSSB1, it has also been shown to self-oligomerise as well as interact with and being modified by several proteins highlighting the versatility that hSSB1 displays in the context of DNA repair. A detailed structural understanding of these processes will likely lead to the designs of tailored hSSB1 inhibitors as anti-cancer drugs in the near future.

## 1 Introduction

Damage to the genetic code must be repaired quickly and efficiently in order to prevent genomic instability. Cellular DNA is under constant threat from both endogenous and exogenous factors, with each cell experiencing tens of thousands of damage events each day [[1], [2], [3]]. This damage must be repaired with high fidelity for the preservation of the genetic and epigenetic code. Failure to protect the DNA can results in the loss or alteration of gene sequences, which in turn can alter protein structure, function and expression; potentially leading to disease states such as cancer and neurodegenerative disorders [4,5]. To protect the genetic code cells have evolved efficient DNA repair pathways that can detect, signal and repair the genome. There are five primary repair pathways, Mismatch repair (MMR), Base Excision Repair Pathway (BER), Nucleotide Excision Repair Pathway, Homologous Recombination Pathway and Non-homologous End-joining Pathway. Each repair pathway specialises in a particular form of DNA damage, although there is a degree of substrate overlap [[6], [7], [8]].

One common element of DNA damage and repair is the presence of single-stranded DNA (ssDNA) which occurs during the processing by repair proteins. This ssDNA is vulnerable to further damage or digestion by nucleases and must therefore be protected. Early in evolutionary life a family of proteins evolved that bind to and protect ssDNA. The single-stranded DNA binding (SSB) protein family (which is characterised by the presence of a highly structurally conserved oligonucleotide/oligosaccharide binding OB domain) is present in all life forms and is encoded by many viruses indicating the importance of this protein (reviewed in [[9], [10], [11], [12]]).

It was initially thought that humans only had a heterotrimeric Replication Protein A (RPA) family member composed of RPA70, RPA32 and RPA14 [13] and the mitochondrial SSB (mtSSB) encoded within the genome. However, we have described two other functional members of the SSB subfamily in humans [14]. While hSSB1 appears to be ubiquitously expressed in all tissues, hSSB2 expression seems to be restricted predominantly to immune cells and the testes. hSSB1 has been demonstrated to be involved in the repair of double strand DNA breaks, stalled DNA replication forks and oxidised DNA adducts [[14], [15], [16], [17], [18], [19], [20]]. While the role of hSSB2 is not yet clear, it appears to functionally compensate for a loss of hSSB1 in several pathways [21].

**Fig. 1:**
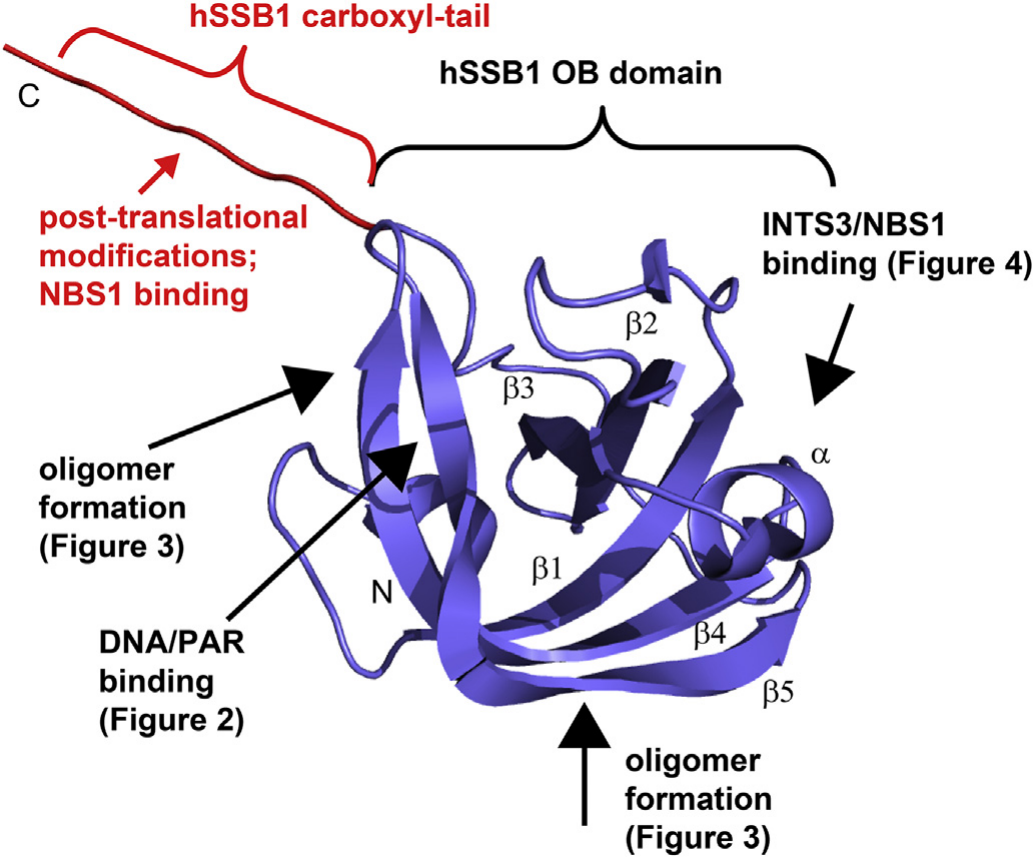
Summary of hSSB1 binding interfaces taken from deposited crystal structures or structural models (PDB ID 4OWX, figshare DOI https://doi.org/10.6084/m9.figshare.3422788 & https://doi.org/10.6084/m9.figshare.4892129) with carboxyl-terminal tail (Nbs1 binding site and location of PTMs) coloured in red.

Most published studies focus on the ssDNA binding ability of hSSB1 (Section 2), however, more recently, hSSB1 has also been shown to be self-oligomerise in the context of oxidative DNA damage repair (Section 3). In addition, both the OB domain and the flexible carboxyl-terminal (C-terminal) tail have been revealed to interact with other important proteins implicated in the maintenance of the genome (Sections 4 and 5). Fig. 1 depicts the structure of hSSB1 and summarises all protein, PAR and DNA binding interfaces discussed in this review.

## 2 DNA Binding of hSSB1

As mentioned above, the most common feature among SSB proteins is the OB domain that binds DNA, RNA, and proteins [[22], [23], [24]] (Fig. 1, blue). While the sequence varies significantly between OB folds from different organisms, these domains share several important structural features [25]: The core is made up of five anti-parallel β-strands organised into a β-barrel structure and a ssDNA binding cleft is formed on one end of the β-barrel whereas the other end is capped by an α-helix (Fig. 1). The length and amino acid sequence of the connecting loops between the β-strands is responsible for the differences in DNA binding specificities of OB domains from different SSBs [25].

The domain organisations of SSBs from several species has been extensively studied over the years. For example, the SSB from *Escherichia coli* (EcoSSB) exhibits a ‘simple’ domain organisation (one sole DNA binding OB domain) that utilises its OB domains to oligomerise into a functional homotetramer [[26], [27], [28], [29]]. In contrast, RPA displays a ‘complex’ domain organisation in which six OB domains spanning across three subunits (RPA70, RPA32 and RPA14) are arranged into a heterotrimer [[30], [31], [32], [33]]. Notably, RPA also engages multiple OB domains for DNA binding, resulting in a significant higher overall affinity compared to hSSB1 (nM versus μM) [34,35].

hSSB1 is a ‘simple’ SSB and exhibits a monomeric state under reducing conditions [12,14,36] in analogy to its archaeal ancestor *Sulfolobus solfataricus* (SsoSSB) that is structurally highly similar to hSSB1 [37,38]. However, under oxidised conditions, hSSB1 can self-oligomerise into homotetramers, which has functional implications in the repair of oxidative DNA damage [17,39,40] (for details see Section 3).

Ren et al. have recently solved the structure of the sensor of ssDNA (SOSS1) complex (composed of hSSB1, INTS3 and C9ORF80) using X-ray crystallography methods, shedding light on how the hSSB1 OB domain binds ssDNA [41]. The crystal structure revealed the structural features of the OB domain: residues 5–109 make up the OB fold with five β-strands (β1, β3, β4, β5 and β6) organised into a β-barrel, and a small α-helix (α1) situated between β3 and β4. An additional small β-strand (β2) is located anti-parallel to β3 [41] (Fig. 1). Importantly, residues 110–211 form an unstructured C-terminal tail [41] that is unable to interact with ssDNA [14]. In contrast, the C-terminal tail of EcoSSB has been shown to play an active role in regulating cooperative binding to ssDNA, however, no direct interaction to ssDNA has been revealed [42].

The DNA binding groove of hSSB1 is located at the N-terminus and lined by residues 2–16, with main contacts between hSSB1 and ssDNA via loops β2-β3 and β4-α1 and strands β4, β5, and β6. ssDNA binding is mediated predominantly via base stacking interactions with W55 and F78, and further contacts are established via electrostatic interactions and hydrogen-bonding contacts involving residues T32, K33, D56, Y74, Y85 and R88 [41]. Interestingly, although no accompanying paper has been published, an additional hSSB1-ssDNA crystal structure has been deposited in the Protein Data Bank (PDB 5D8F) which displays an additional aromatic residue (Y74) stacking with the ssDNA.

**Fig. 2:**
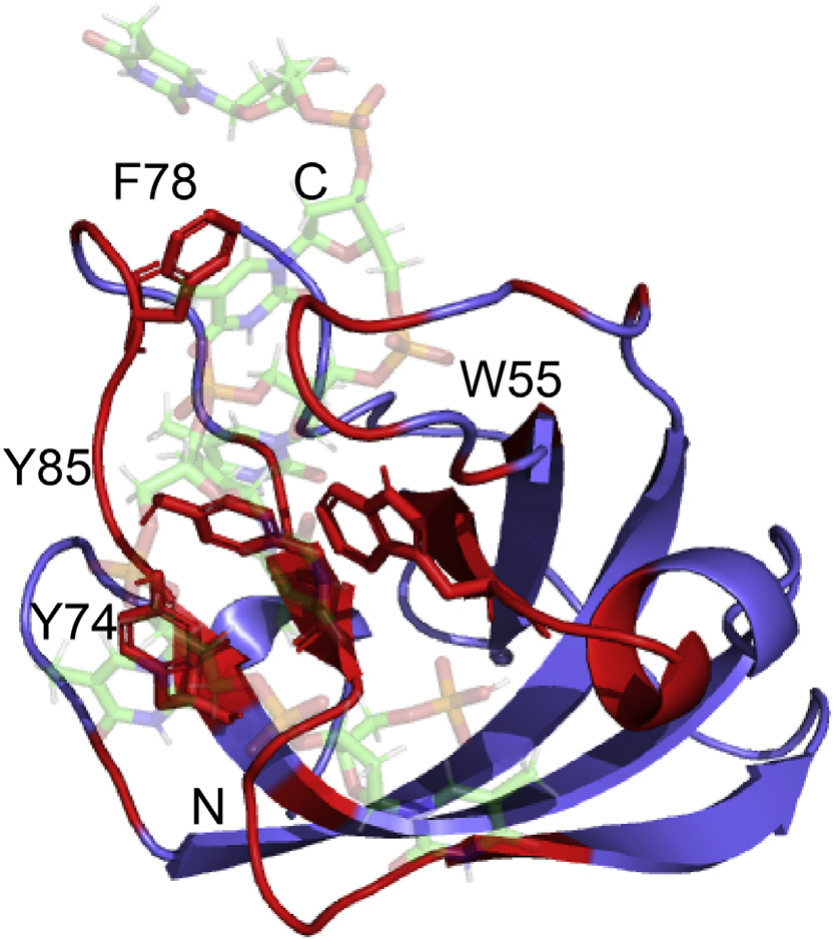
Data-driven structural model of hSSB1-ssDNA complex (figshare DOI https://doi.org/10.6084/m9.figshare.3422788) with DNA (and PAR) binding residues coloured in red and intercalating hSSB1 aromatics indicated (ssDNA in light-green). The orientation of hSSB1 is the same as in Fig. 1.

We have recently determined the solution structure of hSSB1 bound to ssDNA (see Fig. 2 for DNA binding site of hSSB1) and revealed several important differences to the crystal structure [35]. Further, NMR chemical shift mapping carried out by Kariawasam et al., showed considerable shifts in a set of residues not previously recognised as being involved in ssDNA recognition in the crystal structure [36]. Our NMR and biophysical studies have uncovered that recognition of ssDNA in solution is mediated by base stacking with W55, Y74 and F78 in agreement with the deposited crystal structure (PDB 5D8F) as well as an additional aromatic residue (Y85) [35] (indicated in Fig. 2). This was further verified by mutational data from clonogenic survival assays and biolayer interferometry (BLI) studies. The DNA binding interface is conserved between the solution structures of the hSSB1-ssDNA complex and the SsoSSB-ssDNA complex, however, significant differences exists to both crystal structures in relation to the spacing between aromatic residues with respect to the DNA bases [35].

While base-stacking is also a prominent structural feature of DNA binding of both EcoSSB and RPA, respectively, the number and nature of intercalating aromatic OB residues differ from hSSB1 [29,34,35].

## 3 Self-Oligomerisation of hSSB1

Cells are constantly exposed to oxidative stress, which can lead to DNA damage that must be repaired in order to maintain genome integrity [43,44]. Reactive oxygen species (ROS), one of the main sources of oxidative damage, can be generated as a result of exogenous stresses such as ultraviolet (UV) light and produced as by-products of endogenous metabolism [45]. ROS readily react with lipids, proteins and nucleic acids and oxidatively modify them [46]. While modified lipids and proteins can be broken down and resynthesised, the oxidative modification of DNA can compromise the replication and expression of genetic information if not repaired before replication and cell division occur [47].

The oxidation of guanine by ROS results in the formation of 8-oxo-7,8-dihydro-guanine (8-oxoG) [48]. The altered arrangement of hydrogen bond donors and acceptors that result from this modification allow for the modified base (8-oxoG) to form stable Hoogsteen pairs with adenine, in addition to conventional Watson-Crick pairing with cytosine [[49], [50], [51]]. Due to the ability of 8-oxoG to pair with adenine as well as cytosine, a GC to AT transversion may occur during replication as a result of the base modification [52]. As such the accumulation of 8-oxoG in the genome is mutagenic, and the removal of 8-oxoG is crucial in maintaining genomic stability [53,54]. The mechanism responsible for preventing the build-up of 8-oxoG in the human genome is base excision repair (BER).

In humans, the recognition and removal of 8-oxoG base through BER is initiated by human oxoguanine glycosylase (hOGG1) [55,56], an enzyme that possesses two catalytic activities [57]. The hOGG1 enzyme first functions as a DNA glycosylase, cleaving the *N*-glycosidic bond (of a single base in short patch BER and 2–6 bases in long patch BER), then acts as an apurinic/apyrimidinic (AP) nuclease to remove the 3′ phosphate of the resultant abasic site [58]. The removed 8-oxoG base is replaced with guanine by DNA polymerase beta (POLβ) and is ligated in place by the action of DNA ligase III [59].

More recent studies have established that hSSB1 is involved in the removal of 8-oxoG from the genome, playing a central role in the recruitment of hOGG1 to damaged chromatin after oxidative damage [18]. While reduced hSSB1 is a functional monomer, under oxidative conditions the protein has the ability to form homodimers, homotetramers and higher order oligomers [40]. The formation of these hSSB1 oligomers following oxidative stress is vital for the protein to function efficiently in oxidative damage repair as hSSB1 mutants that prevent oligomerisation are unable to efficiently remove and repair 8-oxoG [17,18].

**Fig. 3:**
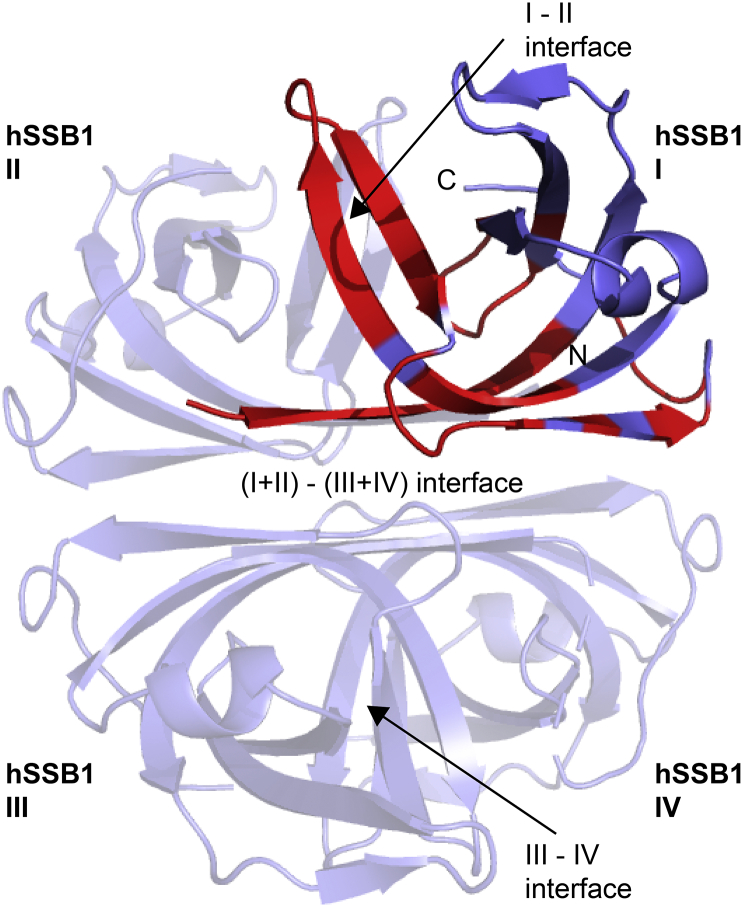
Data-driven structural model of hSSB1 tetramer (figshare DOI https://doi.org/10.6084/m9.figshare.4892129) with oligomer binding residues coloured in red and interfaces between the hSSB1 molecules indicated (hSSB1 molecules 2–4 in light-blue). The orientation of hSSB1 molecule I is the same as in Fig. 1.

We have described a molecular model of the structural details governing the hSSB1 oligomerisation process [40], establishing that hSSB1 can exist as a functional tetramer, with monomer-monomer and dimer-dimer interactions occurring at distinct surfaces of the OB domain, neither of which overlap with the ssDNA binding surface (Fig. 3).

The OB fold of hSSB1 contains three cysteine residues, one of which (cysteine 41; C41) is buried deep inside the hydrophobic core, whereas the remaining two cysteine residues are solvent exposed. These cysteine residues (C81 and C99) facilitate the oxidative damage driven oligomerisation of hSSB1 through the formation of disulphide bonds [40]. In addition to C81 and C99, the OB domain of hSSB1 also contains a set of charged and hydrophobic residues that are also essential for oligomer formation (Table 1) [40]. These residues facilitate the formation of a tetramer (dimer of dimers), in which two dimers sit antiparallel to each other, producing an overall asymmetric tetramer (Fig. 3).

**Table 1:** Key residues in the hSSB1 oligomer formation interface [40].

	Interface between molecules I and II or III and IV (refer to Fig. 3)	Interface between molecules I + II and III + IV (refer to Fig. 3)
Hydrophobic	G13 - M100	L14 - T71
	L14 - L19	G89 - G89
	L17 - L17	I20 - I20
	I50 - V77	
	L82-L82	
Electrostatic	K15-D45	T71 - K72
		K72 - D91
		K72 - G89

In addition to our structural model, two crystal structures of a hSSB1 oligomer in which the hSSB1 monomers interact via the C-terminal tail have been deposited into the PDB database in the absence (PDB 5D8E) and presence of ssDNA (PDB 5D8F) (see also Section 2), respectively, however, no accompanying paper has been published.

Whereas the structural features of the OB domain are largely conserved across all domains of life, the quaternary structure varies between different species. Some complex SSBs, such as human RPA, form hetero-oligomeric complexes [[60], [61], [62]]. Other simple SSBs commonly form homodimers (*Deinococcus radiodurans* DrSSB and *Thermus aquaticus* SSB) [63,64], or act as functional monomers (SsoSSB) [37,38,65], however, one study revealed both dimer and tetramer formation in SsoSSB [66].

Like hSSB1, the SSB from *E. coli* (EcoSSB) [27] forms and binds DNA as a functional homotetramer (PDB 1SRU) [[26], [27], [28]]. Commonalities between the oligomeric structure of hSSB1 and EcoSSB include a stretch of charged and hydrophobic residues at the N-terminus of the OB domain (including G13, L14, K15, N16, L17, N18, L19 and I20 in hSSB1), that are involved in intermolecular interactions, participate in continuous β-sheet formation between molecules I and II (Fig. 3) and stabilise the tetramer through a number of electrostatic and hydrophobic interactions. While the nature of interaction varies, the location of residues participating in oligomerisation is conserved between hSSB1 and EcoSSB with several structurally equivalent interactions occurring in EcoSSB and hSSB1. For example, whereas hSSB1 forms disulphide bonds and hydrophobic contacts with C81 and L82, respectively, the charged residues D96 and R89 in EcoSSB exhibit the structurally equivalent role (via electrostatic interactions). Additionally, residues that impact tetramer formation in EcoSSB [26] are positioned similarly to those important in hSSB1 oligomerisation [40].

EcoSSB oligomers bind DNA in two binding modes that are dependent on the monovalent salt concentration. These are the *(EcoSSB)*_*65*_ binding mode, in which long strands of ssDNA interact with all four subunits of the EcoSSB tetramer and the *(EcoSSB)*_*35*_ binding mode, in which the tetramer binds 35 nucleotides using only two of its four subunits [27,67]. The structural mechanism by which hSSB1 oligomers bind to ssDNA and 8-oxoG base-containing DNA in particular, however, remains to be determined.

## 4 Post-Translation Modifications of hSSB1

The OB fold is a highly conserved construct across all three domains of life and specifically binds ssDNA [14,68] (for details see Section 2), while the flexible C-terminal tail can be post-translationally modified and participates in the interaction with numerous proteins (see Fig. 1, coloured in red).

For example, hSSB1 (along with a number of other essential proteins involved in DNA damage response pathways including DNA-dependant protein kinase DNA-PK, NBS1 and Mre11) is a substrate of the ataxia telangiectasia mutated (ATM) kinase [15,69]. This phosphorylation at threonine 117 (Table 2), following exposure to ionising radiation (IR), is essential for its stabilisation, preventing the degradation of hSSB1 by proteasomes after an IR event [14].

**Table 2:** Post-translation modifications of hSSB1.

	Initiated by	In response to	Consequence
Phosphorylation			
T117	Ataxia telangiectasia mutated (ATM) kinase [14]	Ionising radiation (IR) and IR induced Double stranded breaks	Stabilises hSSB1 preventing degradation by proteasome [14] and Extends the hSSB1 signal away from initial foci [39]
S134	DNA-dependent protein kinase (DNA-PK) [15]	DNA damage due to Replication fork inhibition i.e. replication fork stall/slow progression	Promotes hSSB1-mediated cell survival in response to DNA damage promoting genomic stability [14,15]
Acetylation			
K94	p300 (E1A-accociated protein p300) Histone acetyltransferase [70]	DNA damage events following IR exposure	Acetylation of hSSB1 inhibits ubiquitination and thus ubiquitin-mediated degradation

Phosphorylation of hSSB1 serine residue 134 by DNA-PK (Table 2), a modification that under normal physiological conditions is suppressed by PPP-family serine/threonine phosphatases, is initiated in response to replication fork disruption [13,14]. The effect of this response is unknown, however, it has been established that phosphorylation at this site promotes hSSB1-mediated cellular survival after deleterious events [15] such as DBS formation and replication stress.

As well as phosphorylation in response to DNA damage events, the C-terminal tail of hSSB1 is also acetylated at lysine residue 94 (Table 2) by the histone acetyltransferase p300 [70]. This modification protects hSSB1 from ubiquitination during damage events supporting p300 acetylation of tumour suppressor p53. As a consequence of the hSSB1-p300-p53 interaction (see also Table 3) transcriptionally activation of the p53 target gene p21 [71] takes place. hSSB1 also binds directly to and protects p21 from ubiquitin-mediated degradation [72]. p21 is a cyclin kinase inhibitor that, in the event of DNA damage, works as a cell cycle check point inhibitor, inducing cell cycle arrest [71,72], a mechanism important for ensuring the integrity of the genome before cell division.

**Table 3:** hSSB1 binding proteins and other molecules.

Protein/other molecule	Binding site	Function	Effect	Mechanism
p21	N-tail (residues 1–90)	Cyclin-dependant kinase inhibitor	Protects p21 of ubiquitination and ubiquitin-mediated degradation by proteasomes ensuring adequate cell cycle progression and DNA damage checkpoint activation	hSSB1 acts as a cellular chaperone to p21 preventing degradation [72]
p53	Core domain (100−300)	Tumour suppressor protein	Activates p53 and protects against ubiquitin-mediated degradation in DNA damage events [71]; activated p53 induces expression of p21	hSSB1 allows acetylation stabilisation of p53 at lysine residue 382; inhibiting MDM2-mediated ubiquitination
p300	Not known	Histone acetyltransferase	hSSB1-mediated regulation of p53 acetylation	Acetylates both hSSB1 (see also Table 2) and p53
INTS3 (SOSS complex)	N-tail (residues 1–500)	RNA polymerase II C-terminal domain binding factor participating in the 3′ processing of small nuclear RNAs (snRNA) [76]	INTS3 binding within the OB fold of hSSB1 stabilises and regulates recruitment to ssDNA after damage [75]	INTS3 acts as a scaffold to bridge hSSB1 and C9ORF80 (SSOS formation) [73] and stabilises the complex at DNA damage sites [74]
NBS1 (MRN complex)	N-tail (residues 1–221) [19]	NBS1 is part of the MRN complex associated with DSB repair	hSSB1 in complex with MRN localising to a DSB site is essential for HR repair mechanism	NBS1 links hSSB1 to the MRN complex guiding the protein to the site of a DSB [19,20]
PAR	Entire molecule	Signalling and docking station for DDRs at/adjacent to DSB site	Interaction of PAR and hSSB1 directs hSSB1 to DSB site	hSSB1 simultaneously binds PAR and INTS3 (SOSS1) establishing DDR cascades initiation [68]

## 5 hSSB1 Binding to Other Proteins and Molecules

In addition to post-translational modifications of the C-terminal tail, the hSSB1 monomer also exists in complex with numerous other proteins involved in the detection and repair of DNA single and double-stranded breaks (Table 3). For example, two such complexes are the Mre11-NBS1-Rad50 (MRN) complex and the SOSS1 complex (see also Section 1). Both the MRN and SOSS1 complexes play essential roles in homologous recombination-dependent DSB repair [73].

**Fig. 4:**
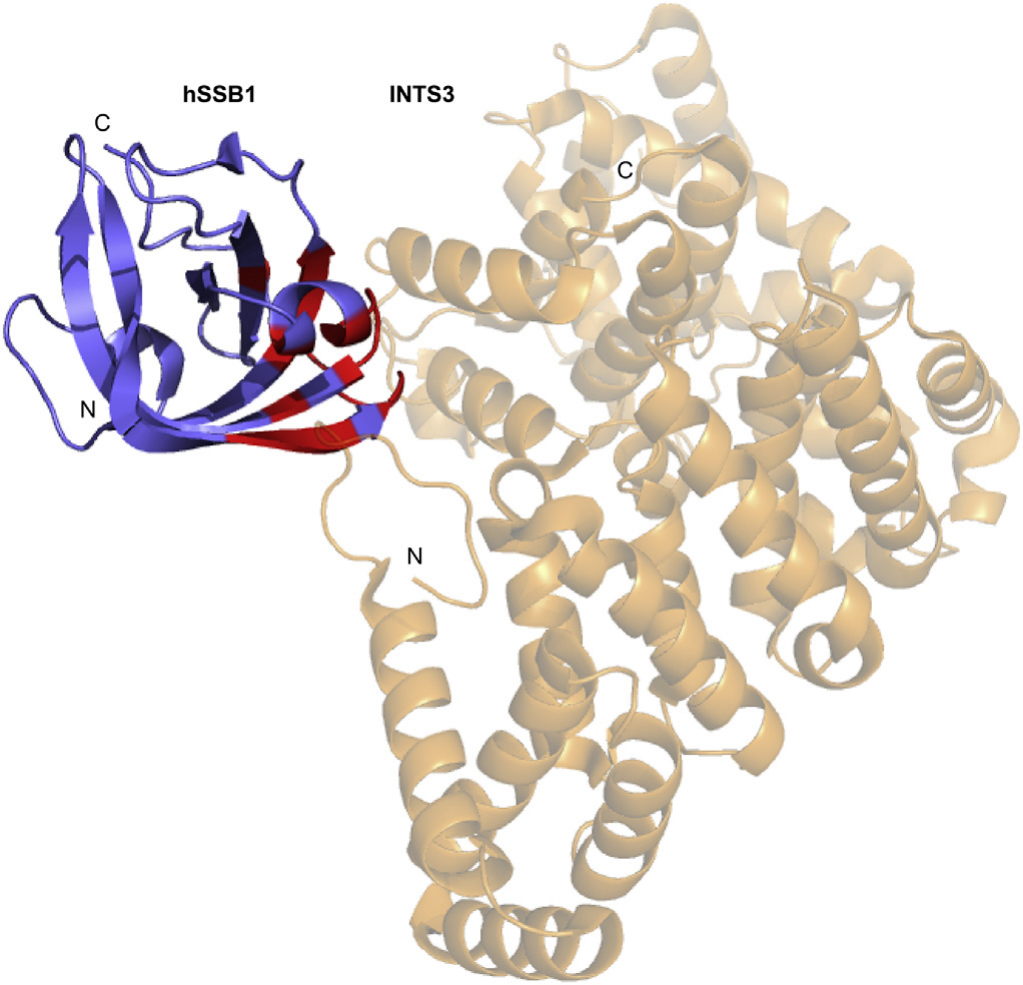
Crystal structure of hSSB1-INTS3 complex taken from PDB ID 4OWX with INTS3 binding resides coloured in red (INTS3 in light-orange). The orientation of hSSB1 is the same as in Fig. 1.

Within the SOSS1 complex (composed of hSSB1, INTS3 and C9ORF80) [74,75] hSSB1 binds directly to INTS3 via its OB domain but not to C9ORF80 [41] (Fig. 4, Table 3). This interaction has been demonstrated to take place independently of DNA damage with constitutive levels consistently detected [74,76]. hSSB1 binding to INTS3 is achieved via two primary interfaces: Interface 1 consisting of a C-shaped cavity within the N-terminal of INTS3 that connects with helix α1 and strand β6 of hSSB1 and interface 2 made up of α17 and α18 and the connecting loop of INTS3 that interacts with strand β1, the α1-β5 loop as well as the C-terminal tail (residues 97–102) of hSSB1 (Fig. 4) [41].

In the case of the MRN complex, two interaction interfaces with hSSB1 have been proposed (Table 3). Firstly, the region of, and surrounding, the BRCT1 domain (residues 111–197) of NBS1, including residues I171 and R215, has been described as the interaction point for hSSB1 via its flexible C-terminal extension (residues 154–211) [20,77]. In contrast, another study [74] has revealed that hSSB1 binds the MRN complex via INTS3 (as part of the SOSS1 complex), which directly interacts with NBS1.

Both the BRCT1/2 domains of NBS1 and the OB domain of hSSB1 also function as poly(ADP ribose) (PAR) binding proteins [68] (Table 3, Figs. 1 and 2). Mechanistically, within seconds of DNA damage detection PAR polymerases induce PAR formation at, or adjacent to the lesion [78,79]. PAR serves as a signal for the recruitment of DNA damage repair (DDR) complexes containing hSSB1. Zhang et al. demonstrated that the hSSB1 OB fold simultaneously binds PAR and INTS3 (SOSS1) establishing a DDR cascade [68].

## 6 Concluding Remarks

hSSB1 has been established as a major player in the maintenance of genome stability. Both the structured OB fold as well as the flexible C-terminal tail has been shown to bind single-stranded DNA and numerous important proteins essential in the DNA repair response. This review has focused on the molecular details of these interactions and describes the underlying structural and biophysical mechanisms. Both NMR and crystallography approaches will be used in the near future to get an even closer insight into the structural basis of hSSB1 action in the context of DNA repair.

Targeting DNA repair pathways has been a widely used strategy for the development of novel cancer drugs for many years (see for example a very recent special issue of the *Cancers* journal about the DNA repair pathways on cancer biology and therapy [80]) as DNA repair is normally upregulated in cancer cells. In cases where DNA damage is artificially induced via classic chemotherapy this higher DNA repair activity needs to be effectively counteracted.

For these reasons, a thorough understanding of the structural details of hSSB1 is an important prerequisite for the future designs of tailored hSSB1 inhibitors as potential anti-cancer drugs that selectively block DNA repair.
